# 2-[^18^F]Fluoropropionic Acid PET Imaging of Doxorubicin-Induced Cardiotoxicity

**DOI:** 10.1007/s11307-024-01978-y

**Published:** 2025-01-14

**Authors:** Juan A. Azcona, Anja S. Wacker, Chul-Hee Lee, Edward K. Fung, Thomas M. Jeitner, Onorina L. Manzo, Annarita Di Lorenzo, John W. Babich, Alejandro Amor-Coarasa, James M. Kelly

**Affiliations:** 1 Department of Radiology, Weill Cornell Medicine, 413 E 69th Street, Room BB-1604, New York, NY 10021 USA; 2 Citigroup Biomedical Imaging Center, Weill Cornell Medicine, New York, NY USA; 3 Department of Pathology and Laboratory Medicine, Weill Cornell Medicine, New York, NY USA; 4 Sandra and Edward Meyer Cancer Center, Weill Cornell Medicine, New York, NY USA; 5 Department of Radiology, Albert Einstein College of Medicine of Yeshiva University, New York, NY USA; 6 Present Address: Ratio Therapeutics, Boston, MA USA; 7 Cardiovascular Research Institute, Weill Cornell Medicine, New York, NY USA; 8 Brain and Mind Research Institute, Weill Cornell Medicine, New York, NY USA

**Keywords:** Doxorubicin, Cardiotoxicity, Positron emission tomography, Short-chain fatty acid, Fatty acids, Metabolism, 2-[^18^F]Fluoropropionic acid, Monocarboxylate transporter, AZD3965

## Abstract

**Purpose:**

Treatment of pediatric cancers with doxorubicin is a common and predictable cause of cardiomyopathy. Early diagnosis of treatment-induced cardiotoxicity and intervention are major determinants for the prevention of advanced disease. The onset of cardiomyopathies is often accompanied by profound changes in lipid metabolism, including an enhanced uptake of short-chain fatty acids (SCFA). Therefore, we explored the utility of 2-[^18^F]fluoropropionic acid ([^18^F]FPA), an SCFA analog, as an imaging biomarker of cardiac injury in mice exposed to doxorubicin.

**Procedures:**

Cardiotoxicity and cardiac dysfunction were induced in mice by an 8-dose regimen of doxorubicin (cumulative dose 24 mg/kg) administered over 14 days. The effects of doxorubicin exposure were assessed by measurement of heart weights, left ventricular ejection fractions, and blood cardiac troponin levels. Whole body and cardiac [^18^F]FPA uptakes were determined by PET and tissue gamma counting in the presence or absence of AZD3965, a pharmacological inhibitor of monocarboxylate transporter 1 (MCT1). Radiation absorbed doses were estimated using tissue time-activity concentrations.

**Results:**

Significantly higher cardiac [^18^F]FPA uptake was observed in doxorubicin-treated animals. This uptake remained constant from 30 to 120 min post-injection. Pharmacological inhibition of MCT1-mediated transport by AZD3965 selectively decreased the uptake of [^18^F]FPA in tissues other than the heart. Co-administration of [^18^F]FPA and AZD3965 enhanced the imaging contrast of the diseased heart while reducing overall exposure to radioactivity.

**Conclusions:**

[^18^F]FPA, especially when co-administered with AZD3965, is a new tool for imaging changes in fatty acid metabolism occurring in response to doxorubicin-induced cardiomyopathy by PET.

**Supplementary Information:**

The online version contains supplementary material available at 10.1007/s11307-024-01978-y.

## Introduction

A significant number of cardiovascular disease cases arise due to an earlier treatment of cancer [1]. The treatment of pediatric cancers with doxorubicin, while often successfully eliminating the cancer, leads to cardiac dysfunction later in life for up to 10% of cancer-free patients [2, 3]. Timely diagnosis of this cardiotoxicity can facilitate disease-mitigating treatment and prevent onset of severe dysfunction or even failure. Thus, there is an urgent need for novel methods to detect incipient injury to the heart in cancer patients undergoing treatment.

Long-chain fatty acids (LCFA) act as the primary energy source for the heart, with glucose, lactate, short-chain fatty acids (SCFA), and ketone bodies also serving as metabolic substrates. Doxorubicin induces mitochondrial oxidative stress and dysfunction in cardiomyocytes through its association with cardiolipin, leading to the inefficient oxidation of LCFA [4, 5]. Aberrant LCFA metabolism is a hallmark of cardiac disease given the heart’s reliance on these substrates for energy. This metabolic dysfunction results in the compensatory uptake and oxidation of SCFA [6–8]. SCFA are preferred substrates under these conditions as they rely on different mechanisms for transport than LCFA [9] and are shorter and therefore more oxygen efficient [8]. Recently, the SCFA analog 2-[^18^F]fluoropropionic acid ([^18^F]FPA) was developed to identify explanted prostate and liver cancers in mice [10–13] by positron emission tomography (PET). Subsequent first-in-human [^18^F]FPA PET imaging of a prostate cancer patient included images of the heart where the delineation along the short and long axes confirm that [^18^F]FPA is taken up by this tissue [14].

Despite the importance of SCFA oxidation to metabolically reprogrammed cardiomyocytes in the diseased heart, PET imaging approaches for targeting this pathway have not been pursued. We hypothesized that [^18^F]FPA would be taken up to a greater extent by hearts exposed to doxorubicin in a manner that is distinguishable by PET. To test this hypothesis, we investigated [^18^F]FPA PET as a modality for imaging overt cardiotoxicity caused by doxorubicin with a view towards applying this technique to imaging early-stage subclinical disease. Here, we report that the hearts of mice exposed to doxorubicin took up [^18^F]FPA to a greater extent than the hearts of healthy mice. Furthermore, we developed an optimized imaging protocol for [^18^F]FPA PET and showed that co-administration of the monocarboxylate transporter 1 (MCT1) inhibitor, AZD3965, increased the contrast between cardiac and non-cardiac tissues and significantly reduced the overall tissue radiation absorbed dose.

## Materials and Methods

Full experimental details, including the radiosynthesis of [^18^F]FPA, descriptions of imaging procedures, biodistribution experiments, dosimetry calculations, blood biomarker measurements, and cardiac acyl-CoA synthetase short chain (ACSS) family activity measurements, are available in the Electronic Supplementary Materials.

### Synthesis of [^18^F]FPA

The synthesis of [^18^F]FPA was carried out according to published methods, with small modifications [15]. Briefly, racemic [^18^F]FPA was synthesized in two steps from methyl-2-bromopropionate using no-carrier-added [^18^F]fluoride. The radiotracer was obtained in activities of up to 1.5 GBq at end-of-synthesis and formulated for injection in saline. [^18^F]FPA was obtained in 20–30% non-decay corrected yield and greater than 99% radiochemical purity.

### Mouse Model of Doxorubicin-induced Cardiotoxicity

Ten-week-old male C57BL/6 J mice (*n* = 22) were injected intraperitoneally with 8 × 3 mg/kg of doxorubicin over the course of two weeks, receiving a cumulative dose of 24 mg/kg. The respective controls (*n* = 18) were injected with saline. All animal studies were approved by the Institutional Animal Care and Use Committee at Weill Cornell Medicine.

### microPET/CT Imaging Studies

Mice (*n* = 9–11 per group) were injected intravenously with 9.25–11.1 MBq of [^18^F]FPA in 100–150 μL saline containing either 5 mg/kg AZD3965 or DMSO (4–6 μL). A 60 min dynamic PET acquisition was performed beginning 30- or 60-min post-injection (p.i.) using the Siemens Inveon™ system. Cardiac uptake was determined by image-based quantification using the AMIDE software and expressed as a ratio of percent injected dose and tissue volume (%ID/cm^3^).

### Statistical Analyses

Data illustrations and analyses were performed using the GraphPad Prism 10 software. All data are expressed as means ± S.E.M.. Statistical tests performed include independent t-tests and two-way ANOVA. Tukey post hoc tests were used for multiple comparisons between all groups and Šídák post hoc tests were used for multiple comparisons between experimental groups and their respective controls. A *p*-value < 0.05 was considered to be statistically significant. All *n* values represent individual biological replicates.

## Results

### Fasting Does Not Alter Cardiac Uptake of [^18^F]FPA

**Fig. 1:**
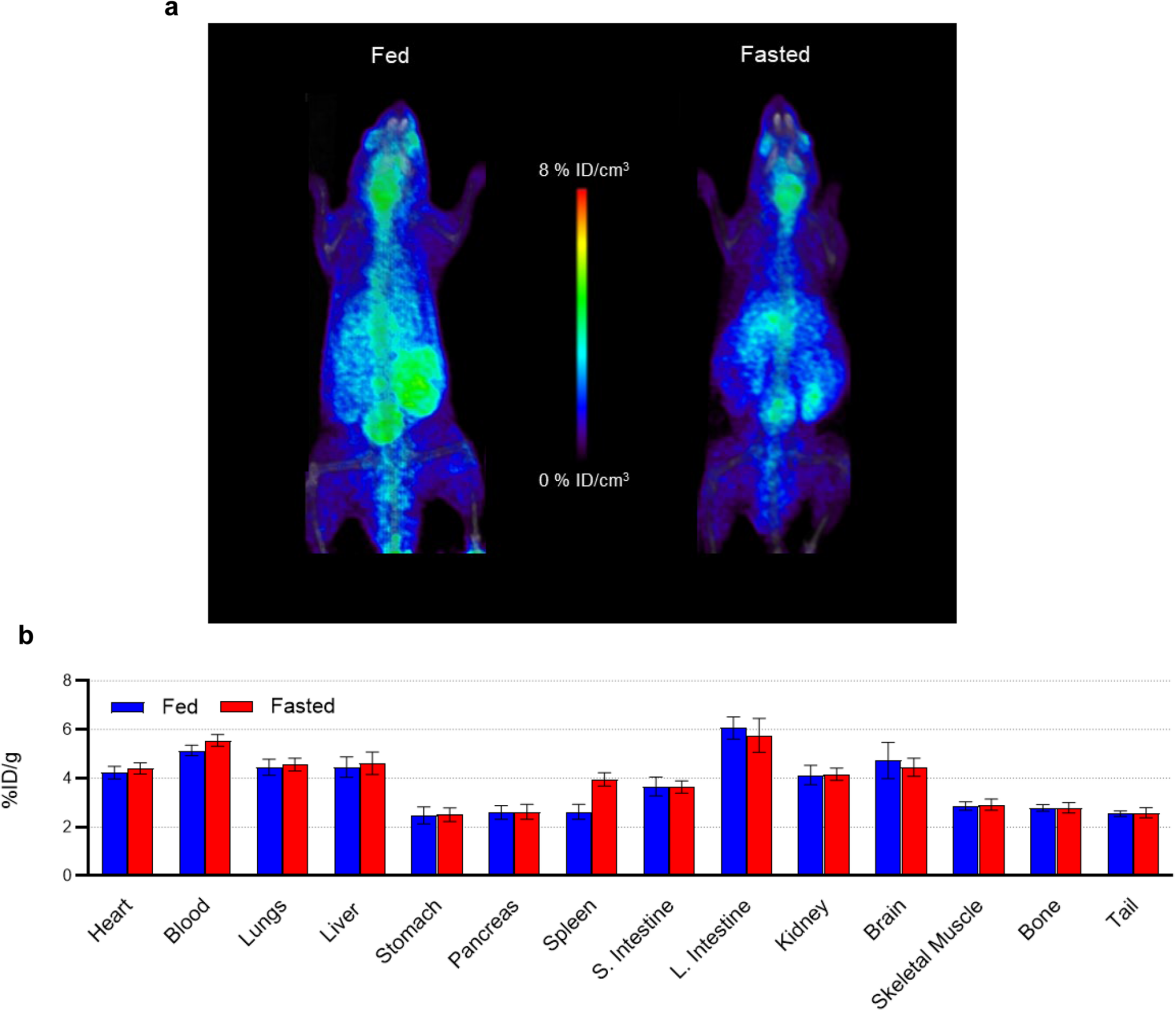
[^18^F]FPA PET/CT Imaging and Tissue Biodistribution of Fed and Fasted Mice. **a** Sixty-minute dynamic acquisitions were performed 60 min p.i. of 9.25–11.1 MBq in fed mice, and mice fasted for 6 h. These animals were euthanized, and their tissues excised 120 min p.i. **b** Tissue uptake was determined as a percent of injected dose per gram tissue (%ID/g) (mean ± S.E.M.; *n* = 4 *p < 0.05, two-way ANOVA, Šídák post hoc).

2-Deoxy-2[^18^F]fluoro-D-glucose ([^18^F]FDG) PET is often preceded by a period of dietary fasting of at least 4–6 h that is critical for some applications of cardiac diagnostic imaging [16]. We therefore assessed whether the cardiac and overall tissue uptake of [^18^F]FPA is influenced by fasting. As shown in Fig. 1, fasting does not significantly affect the uptake of [^18^F]FPA by tissues in healthy mice, although marginal decreases were seen in the brain and the gut.

### AZD3965 Improves Image Contrast for Cardiac [^18^F]FPA PET

**Fig. 2:**
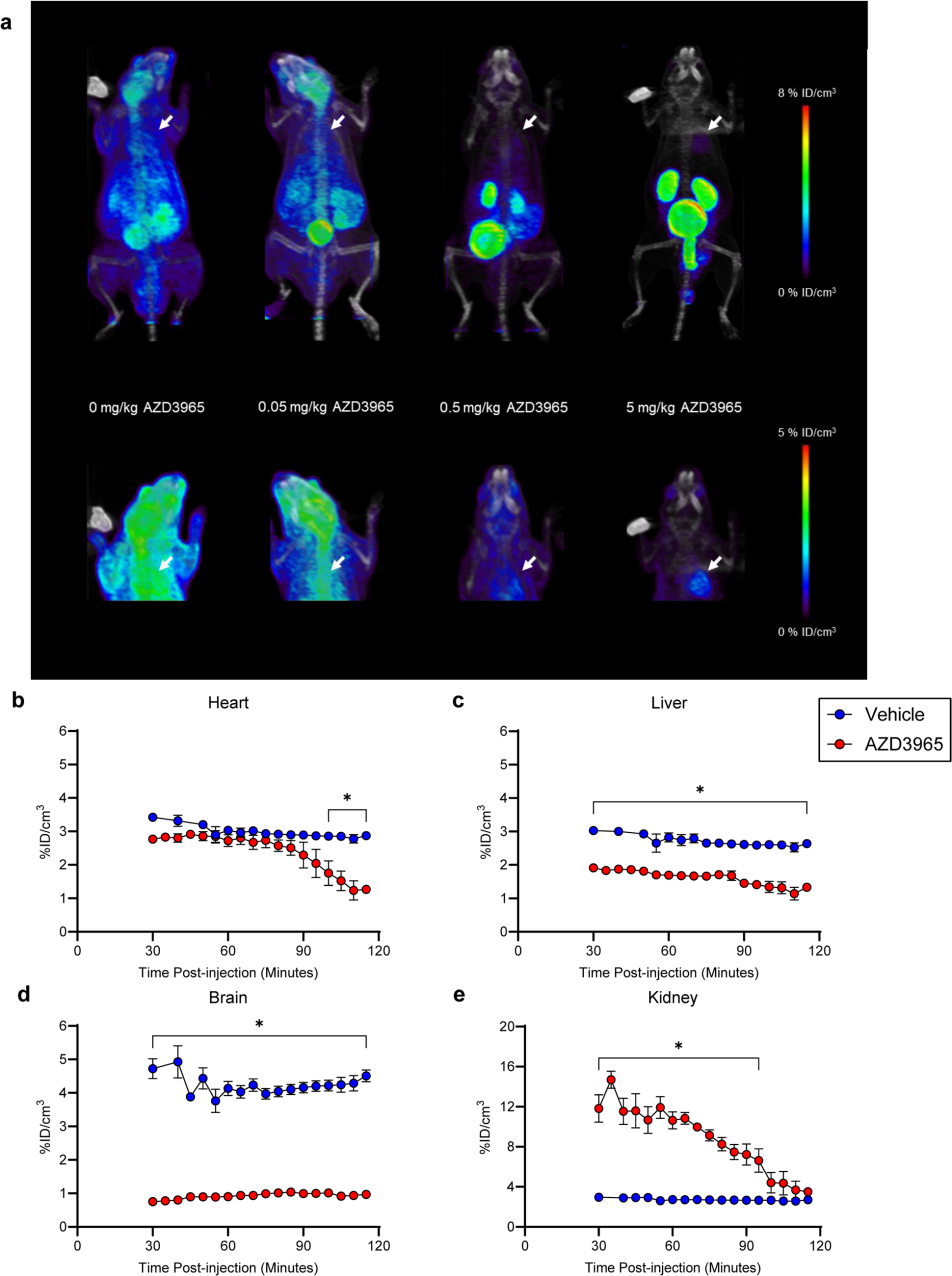
Dose Titration of AZD3965 for [^18^F]FPA PET/CT Cardiac Imaging. **a** Increasing concentrations of AZD3965 (0, 0.05, 0.5, 5 mg/kg) were co-injected with 9.25–11.1 MBq (250–300 μCi) of [^18^F]FPA. The mice were imaged for 60 min by PET/CT starting at 60 min p.i. (*n* = 4). Hearts are indicated by white arrows. Time activity curves for (**b**) hearts, (**c**) livers, (**d**) brains, and (**e**) kidneys taken from image-based quantitation of sixty-minute dynamic PET acquisitions (12 × 5 min frames) starting at 20 and 60 min p.i. are shown. Kinetic differences between animals co-injected with AZD3965 or vehicle (DMSO) are illustrated (mean ± S.E.M.; *n* = 9–11; *p < 0.05, two-way ANOVA, Šídák post hoc).

The uptake of SCFA, such as [^18^F]FPA, by the heart does not primarily rely on MCT1 as it does in other tissues [8] (Supplementary Fig. 1). We therefore reasoned that blocking the ingress of [^18^F]FPA via MCT1 in non-cardiac tissues would improve contrast in the region of the heart and reduce whole body radiation absorbed dose. Indeed, AZD3965 increased the distinction between cardiac and extra-cardiac tissues (Fig. 2a). We treated mice with three concentrations of AZD3965 (0.05 mg/kg, 0.5 mg/kg, 5 mg/kg) and determined that a dose of 5 mg/kg almost completely suppressed [^18^F]FPA uptake in extra-cardiac tissues without decreasing cardiac uptake. Based on time activity curves (TACs) of the hearts, livers, kidneys, and brains of mice injected with [^18^F]FPA and 5 mg/kg AZD3965 or [^18^F]FPA alone, we determined that cardiac uptake of [^18^F]FPA remains essentially unchanged between 30 and 90 min p.i. (Fig. 2b) in both groups. A similar trend was evident in liver and brain, although uptake in these tissues was significantly lower in the AZD3965 group than the DMSO controls (Fig. 2c, d). Kidney [^18^F]FPA uptake is initially increased in the AZD3965 group but rapidly clears from 30 to 120 min p.i. (Fig. 2e). This indicates that AZD3965 promotes urinary clearance of [^18^F]FPA in mice. On the basis of observed trajectories, we identified the optimal window for cardiac imaging to be the interval from 30 to 90 min p.i..

### AZD3965 Reduces Overall Radiation Absorbed Dose

To supplement our image-based assessment of differential tissue radioactivity concentrations in the presence of AZD3965, we measured tissue activities at 2 h p.i. in a biodistribution experiment (Supplementary Table 1). Dosimetry calculations based on these tissue activity data demonstrate that AZD3965 effectively reduces dose to all tissues. These data were extrapolated to humans and translated to a 23% decrease in total body absorbed dose, resulting in a drop from 1.22 × 10^–2^ mSv/MBq to 9.45 × 10^–3^ mSv/MBq for males and from 1.47 × 10^–2^ mSv/MBq to 1.14 × 10^–2^ mSv/MBq for females (Supplementary Table 2). These absorbed doses are comparable to those delivered to patients by [^18^F]FDG [17]. Our calculation assumes a voiding interval of 30 min in line with literature precedent and clinical practice [18]. Longer voiding intervals lead to significantly higher absorbed doses in bladder, and consequently, higher effective doses.

### [^18^F]FPA PET Imaging and Tissue Biodistribution Correlates with Doxorubicin-Induced Cardiotoxicity in Mice

**Fig. 3:**
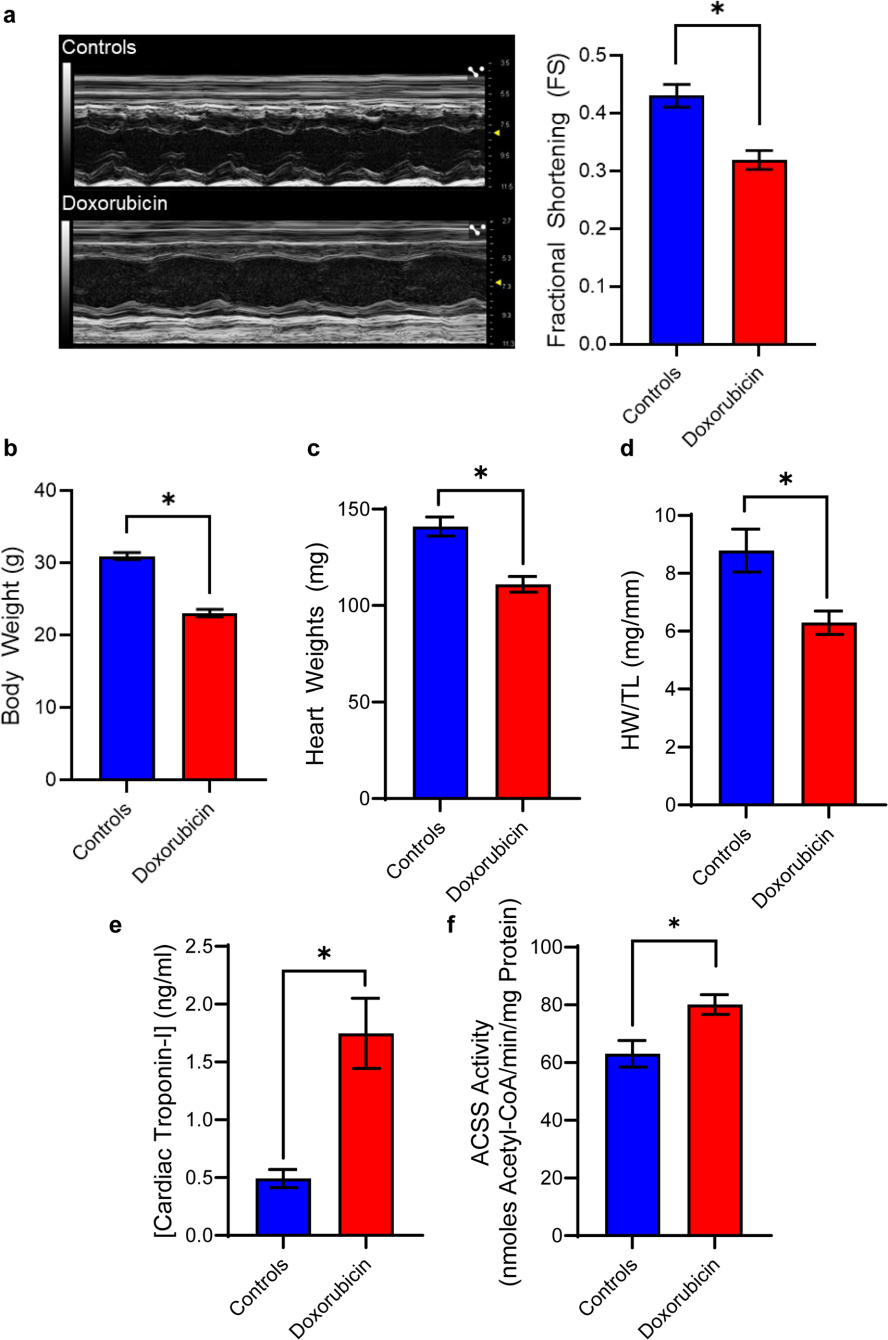
Indicators of Pathology in Mouse Model of Doxorubicin-induced Cardiotoxicity. **a** Doxorubicin-induced cardiac dysfunction was determined by decreased fractional shortening ratios acquired from echocardiograms. (mean ± S.E.M.; *n* = 4–8, *p < 0.05, independent t-test). **b** Mice (mean ± S.E.M.; *n* = 14, *p < 0.05, independent t-test) and (**c**) hearts (mean ± S.E.M.; *n* = 14, *p < 0.05, independent t-test) were weighed post-mortem. **d** Tibia lengths were also measured to generate heart weight to tibia length ratios (HW/TL) (mean ± S.E.M.; *n* = 3–6, *p < 0.05, independent t-test). **e** Serum cardiac troponin-I concentrations were determined by ELISA (mean ± S.E.M.; *n* = 8–10, *p < 0.05, independent t-test) (**f**) Acyl-CoA synthetase short-chain family (ACSS) activity was determined by evolution of pyrophosphate by product in heart homogenates (mean ± S.E.M.; *n* = 9, *p < 0.05, independent t-test).

We confirmed that mice exhibited impaired cardiac function within 8 weeks of the completion of doxorubicin treatment by echocardiography (Fig. 3a). At this timepoint, these mice also exhibited lower body weights and heart weight to tibia length ratios (HW/TL) and elevated cardiac troponin-I levels (Fig. 3b-e). These outcomes are consistent with those found in studies describing doxorubicin-induced toxicity [19, 20] and meet the consensus definition of cardiotoxicity [21]. Another metric for assessing cardiac injury relates to the heart’s propensity for using SCFA as an alternative fuel [6–8]. Cardiac abstraction and metabolism of SCFA are linked to the expression and activities of ACSS enzymes [22], and these are elevated in patients and animals experiencing heart disease [7, 23]. Therefore, we assayed the activities of ACSS enzymes in the isolated hearts of these mice as an index for increased SCFA utilization. As expected, the cardiac tissues from doxorubicin-treated animals exhibited significantly elevated levels of ACSS activities (Fig. 3f). Altogether, these indices indicate biochemical, physiological, and functional changes characteristic of doxorubicin cardiotoxicity.

**Fig. 4:**
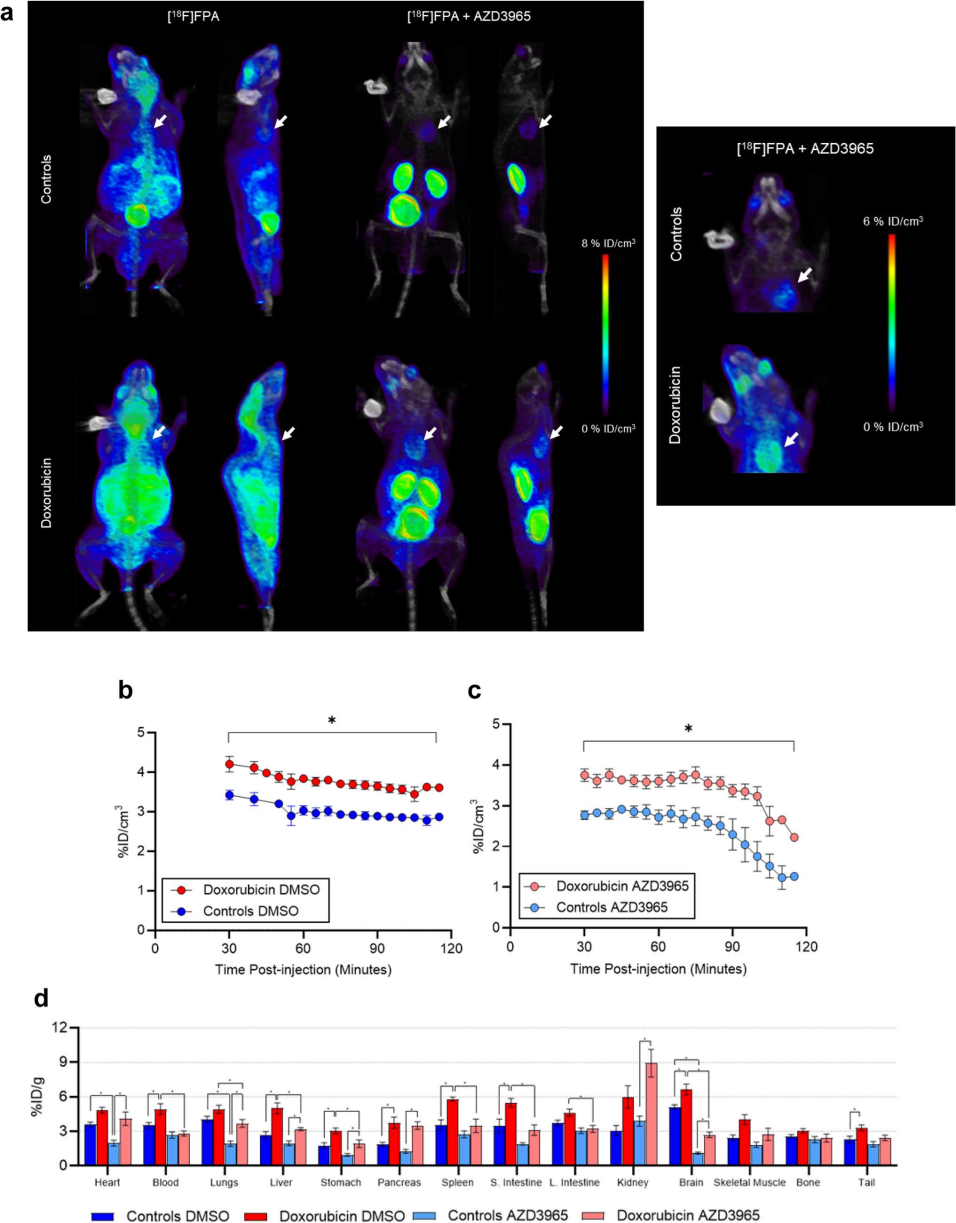
[^18^F]FPA Uptake and PET/CT Imaging in Doxorubicin-induced Cardiotoxicity. Mice were injected with 9.25–11.1 MBq of [^18^F]FPA and 60 min dynamic PET acquisitions (12 × 5 min frames) were performed 60 min p.i.. **a** [^18^F]FPA PET/CT (coronal and sagittal images) of doxorubicin-treated and control mice co-injected with or without 5 mg/kg AZD3965 are shown. Hearts are indicated by white arrows on figures. **b** Cardiac time-activity curves (TACs) in mice administered vehicle (DMSO). TACs were plotted using image-based quantification of injected activity per volume of tissue (%ID/cm^3^) in an ROI drawn over the heart. **c** Cardiac TACs in mice administered AZD3965 (mean ± S.E.M.; *n* = 9–11; *p < 0.05, two-way ANOVA, Šídák post hoc). **d** Ex vivo biodistribution studies were performed 120 min p.i.. The indicated tissues were collected and quantified as a percent of injected dose per gram tissue (%ID/g) by comparison to a standard of known activity. Statistically significant differences between all four variables (controls, doxorubicin, DMSO, AZD3965 groups) are indicated by an asterisk (mean ± S.E.M.; *n* = 5–7; *p < 0.05, two-way ANOVA, Tukey post hoc).

We imaged these mice using [^18^F]FPA PET and observed higher uptakes of [^18^F]FPA in the hearts of mice treated with doxorubicin (Fig. 4a). These differences were even more visually apparent in mice co-injected with AZD3965. We derived TACs from the PET images and determined that animals treated with doxorubicin sustained significantly higher (*p* < 0.05) [^18^F]FPA signals in their hearts across all timepoints of imaging compared to their respective controls (Fig. 4b). Moreover, these statistically significant differences were also apparent in mice co-injected with AZD3965 (Fig. 4c). To further validate our imaging data, we performed a biodistribution study that confirmed increased [^18^F]FPA uptake in the hearts of doxorubicin-treated mice, with the differences even more evident in the groups of mice receiving AZD3965 as a co-injection (*p* < 0.05) (Fig. 4d). [^18^F]FPA uptake at this timepoint was 3.60 ± 0.21%ID/g and 4.85 ± 0.25%ID/g in the untreated mice and 2.00 ± 0.25%ID/g and 4.11 ± 0.58%ID/g in the AZD3965-treated mice, respectively. Doxorubicin also increased [^18^F]FPA uptake in many other tissues, most notably in livers, kidneys, and brains, while co-injection with AZD3965 significantly reduced (*p* < 0.05) [^18^F]FPA uptake in these off-target tissues.

## Discussion

At present, the molecular imaging probes used for cardiac imaging indicate changes in myocardial perfusion [24, 25], ventricular structure and function [26, 27], and metabolism [28–31]. Changes in perfusion and myocardial function are associated with cardiac tissue remodeling and essentially indicate disease that is already evident by other imaging modalities [32]. By contrast, probes which report on changes in cardiac metabolism may be more suitable for detecting pre-symptomatic or sub-clinical disease as metabolic changes occur prior to the onset of cardiac injury [6, 33]. Clinical cardiometabolic PET imaging is dominated by the use of [^18^F]FDG. [^18^F]FDG has the advantages of being readily available in most major hospitals and that its uptake in the heart correlates with some indices of cardiac dysfunction. Nevertheless, cardiac [^18^F]FDG PET imaging is subject to the potentially confounding effect of uptake of this probe by other metabolically active cells. For example, [^18^F]FDG, is avidly taken up by inflammatory cells and is not easily displaced from these cells. The result of this uptake pattern is decreased image sensitivity [34]. Moreover, the first step of glucose metabolism, which is shared by [^18^F]FDG, is its phosphorylation to glucose-6-phosphate. This molecule can undergo glycolysis to afford pyruvate or enter the pentose phosphate pathway. These divergent metabolic pathways may be active to different extents in the diseased heart and may explain the reports of spatial and temporal variability of [^18^F]FDG in hearts with cardiotoxicity [35].

Agents which report on changes in fatty acid metabolism have the potential to directly assess the energetic state of the heart due to this tissue’s reliance on fatty acid oxidation for ATP. Some of the PET tracers developed for this application include [^18^F]fluoro-6-thia-heptadecanoic acid [28], [^11^C]palmitate [31], [^11^C]acetate [29], and [^11^C]lactate [30]. Unfortunately, their imaging applications are limited either by poor retention in the heart or catabolism by other biochemical pathways which do not correlate with the incidence of cardiac disease [28–31]. Of the many alternative substrates used by the LCFA-deficient heart, SCFA are preferentially used as they exploit the existing mechanisms for lipid catabolism [6, 7]. Therefore, we hypothesized that the SCFA analog, [^18^F]FPA, could be used as an indicator of cardiometabolic dysfunction caused by doxorubicin-induced cardiotoxicity.

We began our assessment of [^18^F]FPA as a cardiac imaging agent by analyzing its retention in fed and fasted states. Plasma concentrations of endogenous SCFAs may be influenced by fasting as they are predominantly sourced from the gut microbiome [36]. By contrast to [^18^F]FDG [16], the tissue uptake of [^18^F]FPA is only marginally influenced by a 6 h fasting period. On this basis, we determined that fasting does not suppress cardiac uptake of [^18^F]FPA with the consequence that it is not required before image acquisition. These findings support the practicality of this imaging probe for assessing cardiac disease.

Our experiments in healthy mice indicate that [^18^F]FPA is taken up by nearly every tissue (Fig. 1), which we reasoned could diminish image contrast in the region of the heart and result in undesirable radiation doses to patients. Therefore, we enhanced the contrast to cardiac tissue by co-injecting [^18^F]FPA with AZD3965, an inhibitor of MCT1 [37]. We anticipated this combination would significantly reduce the uptake by non-cardiac tissues reliant on MCT1 for uptake of SCFA [8]. By contrast, SCFA freely enter cardiomyocytes without the need for these transporters [8]. The dose of AZD3965 (5 mg/kg) used to enhance cardiac image contrast may not directly translate to humans as it is several fold greater than the maximal recommended dose for patients (30 mg/70 kg) [38]. However, differences in the pharmacokinetics of this drug are apparent between humans and mice. For example, the therapeutic and subtoxic dose for mice is 100 mg/kg [39]. Since we suppressed background uptake of [^18^F]FPA at 20-fold lesser concentrations of AZD3965 in mice (Fig. 2A), it may be possible to titrate this drug for use in patients to doses substantially below 30 mg.

We tested our approach in a model of doxorubicin-induced cardiotoxicity because treatment of this condition would benefit from the identification of suitable imaging biomarkers for diagnosis and monitoring response to treatment. Cardiac dysfunction caused by doxorubicin is mechanistically and pathologically distinct from more common cardiac diseases which typically lead to cardiac hypertrophy and fibrosis [32]. Therefore, relying on standard physiological and anatomical markers for diagnosis may not be effective for identifying patients at risk of developing disease. [^18^F]FPA uptake was elevated in hearts exposed to doxorubicin and was correlated to increased activities of ACSS. ACSS enzymes are responsible for the thioesterification of SCFA to acyl-CoA intermediates [22]. This is of significance because fatty acyl-CoAs are membrane impermeant [40], which suggests that conversion of [^18^F]FPA to [^18^F]FPA-CoA by ACSS enzymes would lead to its metabolic trapping if it is not subject to further metabolism. Moreover, the activities and expression of this class of enzymes are affected by cardiac disease [7, 23]. Given this class of enzymes contains many isozymes that are typically regulated post-translationally [41], we chose to assay differences in activity rather than protein content. The correlation between ACSS activity and [^18^F]FPA cardiac uptake suggests a mechanistic basis for the increased cardiac uptake of [^18^F]FPA due to doxorubicin-induced cardiac injury.

Doxorubicin induces several potentially pathological changes in mice and humans, including systemic inflammation, weight loss, and cardiotoxicity [3, 20]. These outcomes were achieved in mice with a cumulative dose of 24 mg/kg over 2 weeks. This corresponds to cumulative dose of 72 mg/m^2^ in humans, which is well below the maximum recommended cumulative dose of 450 mg/m^2^ in adult patients [42]. The complex mixture of pathological changes is likely responsible for the increased uptake of [^18^F]FPA (Fig. 4) in blood and non-cardiac tissues. Encouragingly, co-administration of AZD3965 effectively decreased the signal due to [^18^F]FPA in all peripheral tissues of the doxorubicin-treated mice while maintaining cardiac uptake of [^18^F]FPA. Inflamed tissues are likely to use more SCFA as alternative substrates for metabolism or for promoting anti-inflammatory signaling cascades [43]. In this light, the ablation of doxorubicin-induced uptake of [^18^F]FPA in blood, bones, spleens, and tails (Fig. 4d) may reflect inhibition of its uptake by inflammatory cells, which include myeloid populations in the bone, lymphocytes in the spleens, and a combination of both in the blood. In the tails, intravenous injections may be sufficient to stimulate activation of inflammatory cells in the blood which are already primed for activation by doxorubicin [44]. Doxorubicin exposure significantly increased brain uptake of [^18^F]FPA, but this was largely abolished by AZD3965 due to the requirement of MCT1 for permeability of SCFA through the blood brain barrier [45]. The term “chemobrain” is used to describe cognitive dysfunction that can arise during chemotherapy. Cognitive dysfunction is attributed to inflammatory and morphological changes which occur in the brain due to doxorubicin toxicity [46]. These events could be responsible for the increased [^18^F]FPA uptake in the brains of these animals and suggests a possibility for using this tracer to image brain health as it is exposed to doxorubicin. Further study is needed to determine the precise mechanisms which account for all the aforementioned changes in response to doxorubicin, but our observations suggest that AZD3965 renders [^18^F]FPA PET feasible even when systemic inflammation is present.

Interestingly, AZD3965 induces a progressive loss of cardiac signal beginning at 100 min p.i. which is not observed in the absence of the drug (Fig. 2b). These decrements are proportionate in the hearts of both doxorubicin-treated and untreated controls (Fig. 4c) and therefore do not invalidate our comparisons between these groups. One possible explanation for the decreasing signal is that MCT1 serves as an importer of [^18^F]FPA in non-cardiac tissues and an exporter of [^18^F]FPA in the heart. MCT1 is a bi-directional transporter [47] and can participate as an exporter of monocarboxylates in the heart [48]. Inhibition of MCT1 by AZD3965 at early timepoints might therefore serve to trap [^18^F]FPA in cardiac tissue. By 100 min p.i., the available plasma concentrations of AZD3965 may no longer be sufficient to inhibit cardiac efflux of [^18^F]FPA through MCT1 given that the plasma half-life is 2.5 h [39]. In addition, MCT1 inhibition in primary human cardiac myocytes (HCM) promotes a significantly increased uptake of [^18^F]FPA (*p* < 0.05) in these cells (Supplementary Fig. 1). These data support a role for MCT1 for export of [^18^F]FPA from the heart rather than its uptake. Nevertheless, the stable cardiac signal from 30–90 min p.i. affords a broad imaging window that can be readily implemented for clinical scans.

Although we employed a well-characterized model of doxorubicin-induced cardiotoxicity for the evaluation of cardiac [^18^F]FPA PET, we anticipate this imaging strategy may be useful in detecting other forms of cardiac injury. An early and pronounced shift in fatty acid metabolism is a common feature of all cardiac diseases [8, 49]. Consequently, we plan to investigate [^18^F]FPA as a probe for detecting incipient cardiac failure across the spectrum of heart disease, not only the disease arising from cardiotoxicity. We anticipate that our approach will also be translatable to clinical populations. Prior human studies with [^18^F]FPA [14] and AZD3965 [38] in other indications confirm their safety.

One major limitation of our study is that we investigated [^18^F]FPA PET when cardiac dysfunction, as evidenced by decreased fractional shortening, was already evident. At this stage of disease, diagnostic imaging is possible using non-nuclear modalities and cardiac damage is irreversible. Therefore, our future work will investigate the utility of [^18^F]FPA PET as an early indicator of doxorubicin-induced cardiotoxicity. In addition, we found, as have others, that female C57BL/6 mice are not as susceptible to doxorubicin toxicity as males [50]. Although observations in clinical populations support the hypothesis that cardiometabolic changes occur similarly in men and women, we were unable to test this hypothesis in our model.

## Conclusions

We developed an optimized cardiac imaging protocol for [^18^F]FPA PET based on the co-administration of AZD3965, an MCT1 inhibitor currently undergoing clinical evaluation, and demonstrated the suitability of this approach for imaging metabolic changes in the hearts of mice exposed to doxorubicin. As we confirmed that these mice displayed canonical features of cardiotoxicity, these results support the use of [^18^F]FPA and AZD3965 to image doxorubicin-induced cardiotoxicity by PET. Our future studies will be focused on applying this method for imaging other cardiac diseases which are characterized by changes in fatty acid metabolism.

## Supplementary Information

Below is the link to the electronic supplementary material.

Supplementary file1 (DOCX 52 KB)

## Data Availability

All research data is available from the corresponding author upon request.
