# Supplementary material for: 2-[18F]Fluoropropionic Acid PET Imaging of Doxorubicin-Induced Cardiotoxicity
Source: Mol Imaging Biol. 2025 Jan 14;27(1):109–19. doi: 10.1007/s11307-024-01978-y (PMC11805620; doi:10.1007/s11307-024-01978-y)
Supplement: Supplementary file 1 — Supplementary file1 (DOCX 52 KB) [file 11307_2024_1978_MOESM1_ESM.docx]

**Electronic Supplementary Material**

**2-[^18^F]Fluoropropionic Acid PET Imaging of Doxorubicin-induced Cardiotoxicity**

Juan A. Azcona^1^, Anja S. Wacker^1^, Chul-Hee Lee^1^, Edward K. Fung^1,2^, Thomas M. Jeitner^1^, Onorina L. Manzo^3^, Annarita Di Lorenzo^3^, John W. Babich^1,2,4,a^, Alejandro Amor-Coarasa^5,a^, James M. Kelly^1,2,4,*^

- 1. Department of Radiology, Weill Cornell Medicine, New York, NY, USA
  2. Citigroup Biomedical Imaging Center, Weill Cornell Medicine, New York, NY, USA
  3. Department of Pathology and Laboratory Medicine, Weill Cornell Medicine, New York, NY, USA
  4. Sandra and Edward Meyer Cancer Center, Weill Cornell Medicine, New York, NY, USA
  5. Department of Radiology, Albert Einstein College of Medicine of Yeshiva University, New York, NY, USA

1. Present Address: Ratio Therapeutics, Boston, MA, USA

**Corresponding author:**

James M. Kelly, Ph.D.

413 E 69^th^ Street, Room BB-1604

New York, NY, USA, 10021

Phone: +1-(646)-962-6791

Email: [jak2046@med.cornell.edu](mailto:jak2046@med.cornell.edu)

**Materials and Methods**

*General*

Doxorubicin hydrochloride was purchased from Tocris Bioscience, USA and used without further purification. It was dissolved at a concentration of 0.75 mg/mL in sterile saline for injection (Hospira, USA) with the aid of sonication. The solution was stored in the dark at -20 °C for up to 24 h before use. AZD3965 was purchased from MedChem Express, USA and used without further purification. It was prepared as a stock solution in dimethylsulfoxide (DMSO) at a concentration of 25 mg/mL.

*Mouse Model of Doxorubicin-induced Cardiotoxicity*

Eight-week-old male C57BL/6J mice (*n* = 40) were purchased from The Jackson Lab (Bar Harbor, USA). The mice were allowed to acclimate to the vivarium for 10 days prior to intraperitoneal administration of 8 x 3 mg/kg of doxorubicin over the course of two weeks. The mice (*n* = 22) received a cumulative dose of 24 mg/kg. The respective controls (*n* = 18) were injected with saline. The animals were allowed to eat ad libitum and were housed in a vivarium with 12 h alternating light/dark cycles. The PET and biodistribution studies were conducted after cardiac dysfunction was apparent by echocardiography (8 weeks following the completion of dosing). All animal studies were approved by the Institutional Animal Care and Use Committee at Weill Cornell Medicine.

*Cardiac Troponin-I ELISA*

Sera were extracted from whole blood by centrifugation (1,500 x g) and analyzed for cardiac troponin-I (ng/mL) using a mouse cardiac troponin-I ELISA kit (CTNI-1-HS, Life Diagnostics, Westchester, PA).

*Echocardiography*

Imaging was performed using VisualSonics Vevo 770 and 3100 imaging systems as described by Sasset *et al*.^60^. Dimensions of left-ventricle end-diastolic (LVDd) and end-systolic (LVDs) were measured using M-mode traces and applied to calculate fractional shortening (FS) = (LVDd-LVDs)/(LVDd). Diastolic and systolic measurements were estimated based on points of maximum and minimum cavity measurements, respectively^61^.

*Synthesis of [^18^F]FPA*

The synthesis of [^18^F]FPA was carried out according to published methods, with small modifications^15,62^. [^18^F]Fluoride (no-carrier-added, 3.0-5.5 GBq) was produced by a ^18^O(p,n)^18^F reaction using a TR19 cyclotron (Advanced Cyclotron Systems, Canada) and was separated from H_2_[^18^O]O by trapping on a QMA light carbonate cartridge (Waters, USA). Following elution with 5 mg K_2_CO_3_/10 mg kryptofix-222 (Millipore Sigma, USA) in a mixture of 800 µL acetonitrile (Millipore Sigma, USA) and 200 µL H_2_O, the resulting K[^18^F]F/K_2.2.2_ complex was dried azeotropically in 3 cycles at 90 °C. To the dried residue was added a solution of 3 mg racemic ethyl 2-bromopropionate (Millipore Sigma, USA) in dry acetonitrile and the reaction was heated at 90 °C for 10 min. Ethyl 2-[^18^F]fluoropropionate was isolated by semi-preparative HPLC following injection onto a Phenomenex Luna^®^ C18(2), 10 µm, 10x250 mm column. Elution of ethyl 2-[^18^F]fluoropropionate (retention time, t_R_ = 9.7 min) was accomplished using a gradient of 0-90% acetonitrile + 0.1% trifluoroacetic acid (TFA) in H_2_O + 0.1% TFA at a flow rate of 8 mL/min. The labeled compound was trapped on two Oasis® HLB Plus LP cartridges (Waters, USA) connected in series, and the ethyl ester hydrolyzed on-cartridge with 0.5 N NaOH by heating for 10 min at 60 °C. [^18^F]FPA was then eluted with H_2_O, the pH of the final product solution was adjusted to 6-7 with 4 N HCl, and the NaCl concentration diluted to 150 mM by the addition of H_2_O. Radiochemical purity (RCP) was greater than 99% as determined by analytical HPLC on a Chirex 3126 (D)-penicillamine, 4.6 x 150 mm column (Phenomenex, USA) applying an isocratic gradient of 1 mM CuSO_4_ at 1 mL/min. The synthesis was completed within 2 h of end-of-bombardment and [^18^F]FPA obtained in 20-30% non-decay corrected yield.

*Small Animal PET/CT*

PET/CT studies were conducted using the Siemens Inveon^TM^ system. Doxorubicin-treated or control mice (*n* = 9-11 per group) weighing 20-30 g were injected intravenously with 9.25-11.1 MBq of [^18^F]FPA in 100-150 μL saline containing either 5 mg/kg AZD3965 or DMSO (4-6 μL). Sixty-minute PET images were acquired in list mode and converted into histograms of 12 x 5 min frames for dynamic analysis. Image acquisitions began 30- or 60-min post-injection (p.i.). In a separate experiment, control mice (*n* = 4) were fasted for at least 6 h before administration of [^18^F]FPA and PET images were acquired using the same protocol. Cardiac uptake was determined by image-based quantification using the AMIDE software^63^ and expressed as a ratio of percent injected dose and tissue volume (%ID/cm^3^).

*Tissue Biodistribution of [^18^F]FPA*

Doxorubicin-treated (*n* = 5-7) and control mice (*n* = 5-7) were administered 9.25-11.1 MBq [^18^F]FPA in saline containing either 5 mg/kg AZD3965 or DMSO (4-6 μL) and euthanized 2 h p.i. by cervical dislocation under isoflurane. Whole blood, hearts, lungs, livers, stomachs, pancreas, spleens, small intestines, large intestines, kidneys, brains, tails, adductor muscles, and tibias were resected from the animals, measured by length (tibia) or weight, and analyzed for counts per minute (CPM) using a Wizard 2 gamma counter (Perkin Elmer, Waltham, MA). Relative tissue counts were determined by ratios of percent injected dose and tissue mass (%ID/g).

*Dosimetry Calculations*

Volumes-of-interest (VOI) were drawn on the first reconstructed frame at 30-35 min p.i. and applied to the entire dynamic image set. Tissue time-activity data (TAC) extracted from the VOIs were corrected for nuclide physical decay and fitted to mono- and bi-exponential decay curves to model biological clearance from the tissues. Model selection was based on the Akaike information criterion. Average time-activity curves were generated from the combined data and time-integrated activity (TIA) was calculated by numerical integration from 0 to 120 min p.i.. Initial activity was assumed to be 0 for all tissues except heart, which contains blood at the maximum concentration immediately after intravenous injection. TIA after 120 min was calculated analytically from integration of the exponential decay curves fitted to the data. Human TIA’s were extrapolated by scaling mouse values based on the ratio of mouse to human organ masses as a function of total body mass. A voiding interval of 30 min was assumed. TIAs were used in dosimetry software OLINDA/EXM 2.2.3 to estimate radiation absorbed doses to organs and total body.

*Acyl-CoA Synthetase Short-Chain Family (ACSS) Activity in Hearts*

Mouse hearts (*n* = 9 per group) were mechanically homogenized on ice using a Benchmark D1000 Homogenizer (Benchmark Scientific, Sayreville NJ) in 1 mL of buffer containing 80 mM Tris (pH 7.8), 50 mM KCl, 1 mM MgCl_2_, 1 mM ATP, 0.2 mM DTT, and Halt^TM^ Protease Inhibitor (ThermoFisher Scientific, Waltham MA) as described by Groot^24,64^. This mixture was further homogenized by sonication (Diagenode, Denville NJ) at 4 °C. ACSS activity of the homogenates was determined by a pyrophosphate colorimetric assay as described by Kuang *et al*^65^. Reaction conditions were previously optimized to achieve linearity (nmols product/min). The reaction was started by adding 20 μL of lysate to a 1 mL reaction mixture for 30 min at 37 °C and stopped by addition of 5M H_2_SO_4_. Pyrophosphate concentrations were determined by interpolating absorbance values to a linear plot generated using known concentrations of sodium pyrophosphate (Millipore Sigma, USA). Relative ACSS activity was measured as nmol of pyrophosphate generated per minute per mg of lysate protein. Protein concentrations were measured by bicinchoninic acid assay (ThermoFisher Scientific, Waltham MA).

*Tissue Culture*

Primary human cardiac myocytes (HCM) were cultured in myocyte growth media using conditions recommended by the supplier (PromoCell, Heidelberg, Germany). HCM cultures were maintained at 37°C and 5% CO_2_ in a humidified incubator and grown to confluency in 24-well plates before experiments. Passage numbers 4 were used.

*[^18^F]FPA Uptake Assay in HCM*

HCM were incubated in basal myocyte media (200 μL) containing 925 kBq/well [^18^F]FPA, 100 nM of AZD3965 or vehicle (DMSO), and 0.05% bovine serum albumin (BSA) for 1, 2, 4, 8, 16, and 32 min. DMSO was added at a final concentration of 0.1% in volumes of media. “Zero” timepoints were conducted by immediately removing incubation media following addition. After the incubation period, the media was removed, and the cells were washed twice with 1 mL of 1x PBS. The first wash was immediately removed after addition. The cells were incubated with the second wash for a period of 1 min to minimize [^18^F]FPA background signal. Lysates were prepared in two stages: first we detached cells from their substratum using 400 μL 0.5% Trypsin-EDTA, and second, by adding 100 1 N NaOH after the cells had detached. The lysate (500 μL) was aliquoted into counting tubes and analyzed for CPM using a Wizard 2 gamma counter (Perkin Elmer, Waltham, MA). Percent uptake was determined as a ratio of counts in lysates to total counts present in the incubation media. Consistent cell counts were ensured by measuring cell protein concentrations in wells using the bicinchoninic acid assay (ThermoFisher Scientific, Waltham MA). The standard deviations of the measured protein concentrations did not exceed 4% of the means.

**Results**

**Supplementary Figure 1.** MCT1 Inhibition on the Kinetics of [^18^F]FPA Uptake in Primary Human Cardiomyocytes. The graph shown illustrates [^18^F]FPA uptake of HCM as a percent of added activity at 0, 1, 2, 4, 8, 16, and 32 min after the addition of [^18^F]FPA containing AZD3965 (100 nM) or the vehicle (DMSO) control (mean ± S.E.M.; n = 5 *p < 0.05, two-way ANOVA, Šídák post hoc).

**Tables**

**Supplementary Table 1.** Mouse dosimetry of [^18^F]FPA co-administered with AZD3965 or vehicle (DMSO)

| **30 g Male Mouse** | Tissue Activity (%ID/g) | | Absorbed Dose (mSV/MBq) (assuming 2 h voiding interval) | | Absorbed Dose (mSV/MBq) (assuming 30 min voiding interval | |
| --- | --- | --- | --- | --- | --- | --- |
| Target Organ | [^18^F]FPA | [^18^F]FPA + AZD3965 | [^18^F]FPA | [^18^F]FPA + AZD3965 | [^18^F]FPA | [^18^F]FPA + AZD3965 |
| Brain | 5.12 ± 0.19 | 1.11 ± 0.09 | 26.8 | 13.0 | 26.8 | 13.0 |
| Large Intestine | 3.75 ± 0.23 | 3.07 ± 0.24 | 13.6 | 11.9 | 13.6 | 11.0 |
| Small Intestine | 3.16 ± 0.21 | 1.92 ± 0.10 | 13.6 | 11.5 | 13.6 | 10.9 |
| Stomach | 1.77 ± 0.25 | 0.96 ± 0.11 | 14.2 | 11.3 | 14.2 | 11.1 |
| Heart | 3.60 ± 0.21 | 2.00 ± 0.25 | 29.8 | 24.0 | 29.8 | 23.9 |
| Kidneys | 3.09 ± 0.43 | 3.89 ± 0.44 | 27.4 | 30.5 | 27.4 | 30.2 |
| Liver | 2.70 ± 0.30 | 1.98 ± 0.21 | 21.1 | 14.8 | 21.1 | 14.7 |
| Lungs | 4.05 ± 0.27 | 1.94 ± 0.24 | 14.7 | 11.6 | 14.6 | 11.5 |
| Pancreas | 1.88 ± 0.18 | 1.27 ± 0.18 | 13.9 | 11.5 | 13.9 | 11.2 |
| Skeleton | 2.56 ± 0.17 | 2.29 ± 0.27 | 13.2 | 10.7 | 13.2 | 10.5 |
| Spleen | 3.57 ± 0.44 | 2.75 ± 0.31 | 13.2 | 10.9 | 13.2 | 10.7 |
| Testes | X | X | 13.2 | 13.1 | 13.2 | 10.6 |
| Thyroid | X | X | 13.3 | 10.7 | 13.3 | 10.6 |
| Urinary Bladder | X | X | 28.0 | 573.0 | 13.6 | 10.9 |
| Total Body | X | X | 14.1 | 14.2 | 14.0 | 11.0 |

1. Tissue activity estimated from counts in tissues collected 2 h p.i. and expressed as percent of injected dose per gram tissue (mean ± S.E.M). Tissues labeled X were not collected.
2. Residence times calculated from average activity at each time point across all animals.
3. Tissue activity at time 0 assumed to be 0 Bq/mL. For heart, initial activity assumed to be 50% ID/g, based on total mouse volume of ~ 2 mL. Mono-exponential fit used to calculate time-integrated activity between time 0 and first data point.
4. For curves where decreasing exponential was not a good model fit, (brain, bladder, AZD heart) no additional efflux assumed, only physical decay beyond last measured timepoint.

**Supplementary Table 2.** Human radiation dosimetry of [^18^F]FPA estimated from a mouse model

| **Absorbed Dose with 30 min Urinary Voiding (mSV/MBq)** | Human Male | | Human Female | |
| --- | --- | --- | --- | --- |
| Target Organ | [^18^F]FPA | [^18^F]FPA + AZD3965 | [^18^F]FPA | [^18^F]FPA + AZD3965 |
| Adrenals | 1.37E-02 | 1.08E-02 | 1.69E-02 | 1.32E-02 |
| Brain | 1.29E-02 | 4.29E-03 | 1.58E-02 | 5.41E-03 |
| Breasts | N/A | N/A | 1.24E-02 | 9.44E-03 |
| Esophagus | 1.23E-02 | 9.32E-03 | 1.24E-02 | 9.44E-03 |
| Eyes | 1.10E-02 | 7.87E-03 | 1.36E-02 | 9.58E-03 |
| Gallbladder Wall | 1.39E-02 | 1.04E-02 | 1.65E-02 | 1.27E-02 |
| Left Colon | 1.43E-02 | 1.11E-02 | 1.74E-02 | 1.36E-02 |
| Small Intestine | 1.45E-02 | 1.15E-02 | 1.62E-02 | 1.28E-02 |
| Stomach Wall | 1.35E-02 | 1.03E-02 | 1.66E-02 | 1.27E-02 |
| Right Colon | 1.41E-02 | 1.09E-02 | 1.74E-02 | 1.33E-02 |
| Rectum | 1.42E-02 | 1.22E-02 | 1.73E-02 | 1.64E-02 |
| Heart | 1.53E-02 | 1.18E-02 | 1.93E-02 | 1.49E-02 |
| Kidneys | 1.50E-02 | 1.66E-02 | 1.82E-02 | 2.01E-02 |
| Liver | 1.07E-02 | 6.96E-03 | 1.34E-02 | 8.76E-03 |
| Lungs | 1.19E-02 | 9.01E-03 | 1.50E-02 | 1.14E-02 |
| Ovaries | N/A | N/A | 1.75E-02 | 1.48E-02 |
| Pancreas | 1.44E-02 | 1.10E-02 | 1.75E-02 | 1.34E-02 |
| Prostate | 1.40E-02 | 1.27E-02 | N/A | N/A |
| Salivary Glands | 1.27E-02 | 9.14E-03 | 1.44E-02 | 1.04E-02 |
| Red Marrow | 1.15E-02 | 8.86E-03 | 1.40E-02 | 1.09E-02 |
| Skeleton | 1.24E-02 | 9.40E-03 | 1.39E-02 | 1.05E-02 |
| Spleen | 1.33E-02 | 1.03E-02 | 1.64E-02 | 1.28E-02 |
| Testes | 1.19E-02 | 9.52E-03 | N/A | N/A |
| Thymus | 1.24E-02 | 9.42E-03 | 1.55E-02 | 1.18E-02 |
| Thyroid | 1.25E-02 | 9.46E-03 | 1.42E-02 | 1.07E-02 |
| Urinary Bladder | 1.41E-02 | 4.07E-02 | 1.50E-02 | 4.61E-02 |
| Uterus | N/A | N/A | 1.73E-02 | 1.65E-02 |
| Total Body | 1.22E-02 | 9.45E-03 | 1.47E-02 | 1.14E-02 |
| Effective Dose | 1.01E-02 | 8.90E-03 | 1.38E-02 | 1.20E-02 |

Human TIA estimated by mass scaling of mouse TIA.

**References**

1. Sasset L, Manzo OL, Zhang Y, *et al*. Nogo-A reduces ceramide de novo biosynthesis to protect from heart failure. *Cardiovasc Res*. 2023;119(2):506-519. doi:10.1093/CVR/CVAC108
2. Mitchell C, Rahko PS, Blauwet LA, *et al*. Guidelines for Performing a Comprehensive Transthoracic Echocardiographic Examination in Adults: Recommendations from the American Society of Echocardiography. *J Am Soc Echocardiogr*. 2019;32(1):1-64. doi:10.1016/J.ECHO.2018.06.004
3. Hongliang W, Kongzhen H, Ganghua T, Tingting H, Xiang L. Simple and efficient automated radiosynthesis of 2‐18F‐fluoropropionic acid using solid‐phase extraction cartridges purification. *J Label Compd Radiopharm*. 2012;55(9):366-370. doi:10.1002/JLCR.2952
4. De Martino M, Daviaud C, Minns HE, *et al*. Radiation therapy promotes unsaturated fatty acids to maintain survival of glioblastoma. *Cancer Lett*. 2023;570:216329. doi:10.1016/J.CANLET.2023.216329
5. Loening AM, Gambhir SS. AMIDE: a free software tool for multimodality medical image analysis. *Mol Imaging*. 2003;2(3):153535002003031. doi:10.1162/15353500200303133
6. Groot PH. The activation of short-chain fatty acids by the soluble fraction of guinea-pig heart and liver mitochondria. The search for a distinct propionyl-CoA synthetase. *Biochim Biophys Acta*. 1975;380(1):12-20. doi:10.1016/0005-2760(75)90040-5
7. Groot PH. Acyl-CoA synthetases in guinea-pig liver mitochondria: Purification and characterization of a distinct propionyl-CoA synthetase. *Biochim Biophys Acta - Lipids Lipid Metab.* 1976;441(2):260-267. doi:10.1016/0005-2760(76)90169-7
8. Kuang Y, Salem N, Wang F, Schomisch SJ, Chandramouli V, Lee Z. A Colorimetric Assay Method to Measure Acetyl-CoA Synthetase Activity: Application to Woodchuck Model of Hepatitis Virus-induced Hepatocellular Carcinoma. *J Biochem Biophys Methods*. 2007;70(4):649. doi:10.1016/J.JBBM.2007.02.008
